# Supplementary material for: Cloud BioLinux: pre-configured and on-demand bioinformatics computing for the genomics community
Source: BMC Bioinformatics. 2012 Mar 19;13:42. doi: 10.1186/1471-2105-13-42 (PMC3372431; doi:10.1186/1471-2105-13-42)
Supplement: Additional file 1 — Supplementary 1 Cloud BioLinux software documentation in the form of a mini, self-contained website. Users need to download and uncompress the .zip file, and open through a web browser the "index.html" file available on the main directory. (ZIP 1823 kb). [file 1471-2105-13-42-S1.ZIP › Cloud-BioLinux-Package-Documentation/docs/mclpipeline.html]

Bio-Linux Software Documentation Pages

Back to search form

## mclpipeline

|  |  |
| --- | --- |
| Name | mclpipeline |
| Description | **mclpipeline** is a generic pipeline for generating and scrutinizing mcl clusterings. It encapsulates a sequence of programs to be run on some input data in order to obtain clusterings and formatted output representing the clusterings, while maintaining unique file names and file name ensembles corresponding with differently parametrized runs. |
| Homepage | http://micans.org/mcl |
| Remote Documentation | http://micans.org/mcl/man/distindex.html |
